# Improving I-ELM structure through optimal addition of hidden nodes: Compact I-ELM

**DOI:** 10.1038/s41598-024-74446-w

**Published:** 2024-10-01

**Authors:** Sunghyo Seo, Jongkwon Jo, Muhammad Hamza, Youngsoon Kim

**Affiliations:** 1 Department of Information and Statistics, Gyeongsang National University, 501, Jinju-daero, Jinju-si, Gyeongsangnam-do Republic of Korea; 2 Department of Bio & Medical Big Data (BK21 Four Program), Gyeongsang National University, 501, Jinju-daero, Jinju-si, Gyeongsangnam-do Republic of Korea

**Keywords:** Compact node, Hidden nodes, Incremental extreme learning machine, And neural networks, Engineering, Mathematics and computing

## Abstract

Incremental extreme learning machines (I-ELMs) can automatically determine the structure of neural networks and achieve high learning speeds. However, during the process of adding hidden nodes, unnecessary hidden nodes that have little relevance to the target may be added. Several studies have proposed methods to overcome this problem by measuring the relevance between hidden nodes and outputs and adding or removing hidden nodes accordingly. Random hidden nodes have the advantage of creating diverse patterns, but they encounter a problem in which hidden nodes that generate patterns with little or no relevance to the target can be added, thereby increasing the number of hidden nodes. Unlike in existing I-ELMs, which use random hidden nodes, we propose a compact I-ELM algorithm that initially adds linear regression nodes and subsequently applies a method to ensure that the hidden nodes have patterns differing from the existing ones. Based on benchmark data, we confirmed that the proposed method constructs a compact neural network structure with fewer hidden nodes compared to the existing I-ELM systems.

## Introduction

Feedforward neural networks (FNNs) have been extensively studied for their ability to approximate nonlinear functions and provide models for phenomena that are challenging to address using traditional statistical methods. The back-propagation algorithm, which is mainly used to train FNNs, adjusts all the connection strengths of the network iteratively during learning, resulting in a low learning speed. Moreover, as it uses the gradient of the gradient descent method to reduce errors, there is no guarantee that it will always converge to the global minimum. Furthermore, back-propagation algorithms that use the gradient descent method suffer from the vanishing gradient problem, where the gradient becomes smaller and approaches zero during learning. To improve these issues with FNNs^1^, proposed a learning algorithm called the extreme learning machine (ELM) for single-hidden-layer FNNs (SLFNs). SLFNs are FNNs comprising L nodes in one hidden layer. ELM is a learning algorithm that randomly determines and fixes the connection strengths of the hidden nodes and then determines the output weights of the hidden nodes using the ordinary least squares (OLS) method. ELMs have been applied in diverse fields owing to their high learning speeds and excellent performances^2– 6^^[,7,8^ and ^9^ indicated that determining the number of hidden nodes in ELMs requires a trial-and-error process, and sometimes additional hidden nodes are required compared to other algorithms. Furthermore^8^ and ^9^ , indicated that underfitting or overfitting can occur if there are too few or too many hidden nodes in an ELM, emphasizing the importance of selecting an appropriate number of hidden nodes when designing the SLFN structure.

 Proposed^7^ an incremental ELM (I-ELM) algorithm to address the problem associated with determining the number of hidden nodes in applications using ELM. I-ELM is an ELM that starts with no hidden nodes and gradually adds random hidden nodes one-by-one. When a new hidden node is added, the output weights of the existing hidden nodes are fixed, and hidden nodes are added until the stopping criteria (i.e., the maximum number of hidden nodes and performance threshold) are met. Though I-ELMs are simple and efficient, they encounter a problem in which new hidden nodes that are added may be similar to previous hidden nodes or play a minor role in affecting the output.

To overcome the problem of an increase in the number of hidden nodes due to the addition of unnecessary hidden nodes in I-ELM^1^, proposed convex I-ELM (CI-ELM), which integrates Barron’s convex optimization^10^ with I-ELM. CI-ELM recalculates the output weights of all hidden nodes using a convex optimization method when a new hidden node is added. Using this method, it achieves a more compact structure with fewer hidden nodes and improved performance than I-ELM^11^. proposed enhanced I-ELM (EI-ELM), which generates multiple candidate hidden nodes and selects and adds the hidden node that reduces the error to the greatest extent^9^. proposed pruned ELM (P-ELM), which combines pruning (a destructive method) with ELM^2^ and ^13^. proposed optimal-pruned ELM (OP-ELM), which calculates the rank of hidden nodes by applying least-angle regression (LARS) to measure the relevance between the hidden nodes and targets and also calculates the prediction sum of squares using the leave-one-out method to remove hidden nodes^14^. proposed Tikhonov regularization optimal-pruned (TROP)-ELM, which calculates the rank of hidden nodes using the L1 norm in the existing OP-ELM system and calculates the output weight of the hidden layer using L2 norm regularization (penalty)^15^. proposed EM-ELM, which adds random hidden nodes one-by-one or in groups to SLFNs^16^. proposed the constructive hidden nodes selection method for ELM (CS-ELM), which focuses on the design of the neural network structure of an ELM. CS-ELM is an algorithm that considers the selection of hidden nodes as a variable selection problem in linear regression models. It selects and determines hidden nodes from many hidden nodes using the stepwise method. Other studies attempted to reduce unnecessary hidden nodes by optimizing their parameters using evolutionary algorithms (EAs) as a global search method^8^. proposed evolutionary ELM (E-ELM), which applies a method to determine the input weights of hidden nodes using differential evolution (DE)^17^, which is a type of EA^18^. proposed self-adaptive evolutionary ELM, which uses the self-adaptive differential evolution algorithm. Likewise^19^, proposed optimized ELM (O-ELM), which determines the input weights of the hidden nodes using a genetic algorithm (GA)^20^. proposed particle swarm optimization (PSO)-ELM, which combines ELM with PSO, a method similar to EA. Han et al. proposed IPSO-ELM^21^, which applies an improved method of PSO, and IPSO-EM-ELM^22^, which combines IPSO with EM-ELM^23^. proposed analytical incremental learning, which calculates the input and output weights of hidden nodes based on a recursive generalized inverse method rather than using random numbers. Consequently, this method can only be used when the inverse function of the activation function exists^24^ and ^25^. sought to obtain a compact neural network by applying orthogonal transformation to the hidden layer output in I-ELM. In addition, TSP based on IPSO-ELM, which optimizes the parameters of the model using synchrophasors using IPSO, was developed by^26^, and OS-Extreme Learning Machine (EOSELM) with binary Jaya (BinJaya)-based feature selection was developed by^27^.

Prior research has endeavored to address the issues associated with I-ELM, wherein novel hidden nodes are incorporated that bear resemblance to preexisting nodes or have negligible impact on the result. The number of hidden nodes increases and the length of learning increases significantly as a result of the addition of these minor hidden nodes. Former studies have used techniques to select the most relevant hidden nodes for the output or remove the nodes which are less relevant^9,11– 14^, and^16^. They have also applied methods to recalculate the output weights of existing hidden nodes when a new hidden node is added^1^ or to generate multiple hidden nodes at the same time. By figuring out the input weights and bias values that affect the functions of random hidden nodes using evolutionary algorithms (EAs) and optimization techniques, researchers have also tried to lower the number of hidden nodes^8,16,17^, and^19– 22^. In order to create a neural network with the right number of hidden nodes, we proposed a compact I-ELM method that makes sure that every hidden node added to the hidden layer has a unique pattern. The Compact I-ELM system starts learning by adding the first hidden node, in contrast to the current I-ELM system, which starts learning with no hidden nodes. The activation function is a linear function with an output weight fixed at 1, and the input weight of the first hidden node is determined by applying OLS. This method uses a linear function for the hidden node’s activation function to add a hidden node corresponding to a linear regression model. In order to make sure that newly added hidden nodes exhibit a different pattern from previously added nodes, they are transformed to be orthogonal to the connection strength of the prior node.

The rest of the paper is organized as follows: ELM and I-ELM is reviewed in Sect. “Existing approaches”. Section "Proposed compact I-ELM approach" comprises of proposed method; Compact I-ELM. Sections “Simulation study” and “Performance evaluation” present the results obtained using benchmark data, and Sect. "Conclusion and recommendation" concludes the study.

## Existing approaches

### Extreme Learning Machine (ELM)

The Extreme Learning Machine (ELM) is a fast learning algorithm of the single-hidden-layer feedforward neural network (SLFNs). It first randomly initializes the bias of the hidden layer neurons as well as the weights between the input and hidden layers. Finally, it computes the weights between the hidden and output layers using the least-squares method^28^.

Equation (1) presents the mathematical model of SLFNs composed of $\:L$ hidden nodes with the activation function $\:g($·) for $\:N$ training data $\:\left\{\left({x}_{j},{t}_{j}\right),\:{x}_{j}\in\:{R}^{n},\:{t}_{j}\in\:R,\:j=\text{1,2},\cdots\:,N\right\}.$

1 $$\sum\:_{i=1}^{L}{\beta\:}_{i}g\left({x}_{j}\right)=\sum\:_{i=1}^{L}{\beta\:}_{i}g\left({w}_{i}\cdot\:{\text{x}}_{\text{j}}+{b}_{i}\right)={o}_{j},\:\:j=1,\:2,\cdot \cdot\cdot,\:N$$

Here, $\:{\varvec{w}}_{\varvec{i}}={\left[{w}_{1i},{w}_{2i},\cdots\:,{w}_{ni}\right]}^{\top\:}$ is a vector representing the input weights of the $\:{i}^{th}$ hidden node and $\:{b}_{i}$ is the bias of the $\:{i}^{th}$ hidden node. $\:{\beta\:}_{i}$ indicates the output weights connecting the $\:{i}^{th}$ hidden node to the output, and $\:{o}_{j}$ is the output for the $\:{j}^{th}$ sample. $\:{w}_{i}\cdot{x}_{j}$ represents the dot product of $\text{w}_i \cdot \text{x}_j$ and $\:{x}_{j}$.

In^1,29^ and ^30^, it was found that if the activation function $\:g$ is infinitely differentiable, it can be proven that the required number of hidden nodes $\:L<N$. Therefore, we may obtain Theorem 2.1.

#### Theorem 2.1

states that any random values and selected by any continuous probability distribution from intervals and, respectively, and given a standard SLFN with hidden nodes and an activation function : R → R, which is infinite over where and ∈ **R**^m^, the probability that the hidden layer output matrix of the SLFN is invertible is one, and it holds that^1^ and ^30^, .

It has been proven that when $\:N$ data points are provided, an SLFN composed of $\:L$ hidden nodes with activation function $\:g($·) can typically approximate the error between the target $\:t$ and neural network output $\:{o}_{i}$ to 0^1] and [31^. Therefore, $\:{\beta\:}_{i}$, $\:{\varvec{w}}_{i}$, and b_i_ exist such that $\:{\sum\:}_{j=1}^{N}{\left|\right|{o}_{j}-{t}_{j}\left|\right|}_{2}\:=\:0$, and Eq. (1) can be written as in Eq. (2) where $\:{\left|\right|{o}_{j}-{t}_{j}\left|\right|}_{2}$ is the Euclidean norm, which is the $\:L2$-norm.

2 $$\:\sum\:_{i=1}^{L}{\beta\:}_{i}g\left({\mathbf{w}}_{i}\cdot\:{\mathbf{x}}_{j}+{b}_{i}\right)={t}_{j},\quad j=1,\cdot\cdot\cdot\;\:N$$

As SLFN assumes $\:L$ hidden nodes with activation function $\:g\left(x\right)$, where $\:{x}_{i}\in\:{R}^{n}$ and $\:{t}_{i}\in\:{R}^{m}$, to learn $\:N$ samples $\:({x}_{i},\:{t}_{i}\:)$. This can be expressed as a matrix in Eq. (3):

3 $$\varvec{H}\varvec{\beta\:}=\varvec{T}$$

where,

$$\:\mathbf{H}({\mathbf{w}}_{1},\cdots:,\:{\mathbf{w}}_{L},\:{b}_{1},\cdots:\:,\:{b}_{L},\:{\mathbf{x}}_{1},\cdots:\:,\:{\mathbf{x}}_{N})\:={\left[\begin{array}{c}g({\mathbf{w}}_{1}\cdot\:{\mathbf{x}}_{1}+{b}_{1})\:\:\:\:\cdots\:\:\:\:g({\mathbf{w}}_{L}\cdot\:{\mathbf{x}}_{1}+{b}_{L})\:\\\:\vdots\:\:\:\:\:\:\:\:\:\:\:\:\:\:\:\:\:\:\cdots\:\:\:\:\:\:\:\:\:\:\:\:\:\:\:\:\:\:\:\vdots\\\:g({\mathbf{w}}_{1}\cdot\:{\mathbf{x}}_{N}+{b}_{1})\:\:\:\cdots\:\:\:\:g({\mathbf{w}}_{L}\cdot\:{\mathbf{x}}_{N}+{b}_{L})\end{array}\right]}_{\:N\times\:L},$$

$$\:\beta\:={\left[\begin{array}{c}{\beta\:}_{1}\\\:\vdots\\\:{\beta\:}_{L}\end{array}\right]}_{\:\:L\times\:1}\:\:,\:\:\:\:\:\:\:and\:\:\:\:T=\:{\left[\begin{array}{c}{t}_{1}\\\:\vdots\\\:{t}_{N}\end{array}\right]}_{\:N\times\:1}$$

Here, $\:\varvec{H}$ is the matrix of the hidden layer outputs, and the $\:{i}^{th}$ column of $\:\varvec{H}$ represents the output of the $\:{i}^{th}$ hidden node for input **x**. β is a vector representing the output weights.

The output weights that link the hidden nodes to the output function are computed analytically by the ELM algorithm after it has arbitrarily determined and fixed the input weights and bias values of the hidden nodes. This algorithm generates random numbers to determine the values of the input weight $\:{w}_{i}$ and bias $\:{b}_{i}$ of the hidden node, respectively. The output matrix $\:\varvec{H}$ of the hidden layer is also kept fixed since the input weights is not changed while learning and is instead left constant. Finding $\:\beta\:$ in linear system $\:\varvec{H}$β = **T** and determining the $\:\widehat{\beta\:}$ value that satisfies Eq. (4) is the same as training an SLFN using ELM. The Eq. (4) is;

4 $$\parallel\varvec{H}\widehat{\beta\:}-T\parallel=\underset{\beta\:}{{min}}\parallel\varvec{H}\beta\:-T\parallel.$$

The ELM algorithm is presented in Algorithm 2.1.

#### Algorithm 2.1

**ELM**.

In order to begin with algorithm 2.1, the activation function is $\:g(.)$, the number of hidden nodes is set to $\:L$, and the training set is defined as $\:\aleph\:=\left\{\right({x}_{j},\:{t}_{j}\left)\:\right|\:{x}_{j}\in\:{R}^{n},\:{t}_{j}\in\:R,\:j=1,\cdots\:,\:N\:\}$. The ELM algorithm may then be summed up as follows:

#### Step (1)

Define the hidden layer node number andomly assign input weights and hidden layer biases for.

#### Step (2)

Calculate the hidden layer output matrix .

#### Step (3)

Determine the output weight using Eq. (5).

If the number of hidden nodes is equal to the number of data points $\:(L=N)$ and an inverse matrix exists, then the hidden layer output matrix $\:H$ is a square and invertible matrix. Therefore, $\:\beta\:$ can be estimated via $\:\widehat{\beta\:}=\:{H}^{-1}T$ by using the inverse matrix of $\:\varvec{H}$. However, if an inverse matrix does not exist, then the inverse matrix of $\:\varvec{H}$ cannot be calculated, and $\:\beta\:$ cannot be estimated using the inverse matrix. In this case, the optimal solution can be obtained with the least square method using the Moore-Penrose inverse function, which is shown in Eq. (5)^31,32^.

5 $$\widehat{\beta\:}={\varvec{H}}^{+}\varvec{T}$$

Here, $\:{H}^{+}$is the Moore-Penrose generalized inverse of matrix $\:\varvec{H}$.

### Incremental-Extreme Learning Machine (I-ELM)

I-ELM is an ELM system that adds random hidden nodes one-by-one. When a new hidden node is added, the parameters of the existing hidden nodes remain fixed and the output weight of the new node is calculated using the same method as in ELM. I-ELM is an ELM that adds hidden nodes during training until the stopping criteria set (i.e., the maximum number of hidden nodes and error threshold) are met.

The I-ELM learning process is as follows. The residual $\:{e}_{i}$ value of the output function $\:{f}_{i}$ and the objective function f for a neural network with $\:i$ hidden nodes is $\:f-{f}_{i},\:({e}_{i}=f-{f}_{i})$. The output weight of the last added $\:{i}^{th}$ hidden node is calculated using Eq. (6):

6 $${\beta\:}_{i}=\frac{\langle{e}_{i-1},\:{g}_{i}\rangle}{{{g\parallel}_{i}}\parallel^{2}}$$

given that $\:\underset{i\to\:+\infty\:}{{lim}}{e}_{i}=0$. $\:{g}_{i}$ is the output of the $\:{i}^{th}$ hidden node and, $\:{e}_{i-1}$ is the residual of the neural network after the $\:{\left(i-1\right)}^{th}$ hidden node has been added before the $\:{i}^{th}$ hidden node is added, and ${e}_{i-1}\cdot{g}_{i}$is the inner product of $\:{e}_{i-1}$ and $\:{g}_{i}$. After the $\:{i}^{th}\:$hidden node has been added, the residual of the neural network become $\:{e}_{i}=f-{f}_{i}=f-({f}_{i-1}+{\beta\:}_{i}{g}_{i})={e}_{i-1}-{\beta\:}_{i}{g}_{i}$.

Considering $\:\varDelta\:\:={\parallel{e}_{i-1}\parallel}^{2}-{\parallel{e}_{i}\parallel}^{2}$ by completing the square, we can determine that ${\beta\:}_{i}=\langle{e}_{i-1},\:{g}_{i}\rangle/{\parallel{g}_{i}\parallel}^{2}$ by simplification:

7 $$\begin{aligned}&\:\varDelta\:\:={\parallel{e}_{i-1}\parallel}^{2}-{\parallel{e}_{i}\parallel}^{2}\\& \:={\parallel{e}_{i-1}\parallel}^{2}-{\parallel{f-(f}_{i-1}+{\beta\:}_{i}{g}_{i})\parallel}^{2} \\ & ={\parallel{e}_{i-1}\parallel}^{2}-{\parallel{e}_{i-1}-{\beta\:}_{i}{g}_{i}\parallel}^{2} \\& ={\parallel{e}_{i-1}\parallel}^{2}-({\parallel{e}_{i-1}\parallel}^{2}-2{\beta\:}_{i}\parallel{e}_{i-1}\parallel\:\parallel{g}_{i}\parallel+{\parallel{g}_{i}\parallel}^{2}) \\& ={2\beta\:}_{i}\langle{e}_{i-1}{,g}_{i}\rangle-{\beta\:}_{i}^{2}{\parallel{g}_{i}\parallel}^{2} \\&={\parallel{g}_{i}\parallel}^{2}\left(\frac{{\langle{e}_{i-1},\rangle}^{2}}{{\parallel{g}_{i}\parallel}^{4}}-{\left({\beta\:}_{i}-\frac{\langle{e}_{i-1}{\:,g}_{i}\rangle}{{\parallel{g}_{i}\parallel}^{2}}\right)}^{2}\right)\end{aligned}$$

By solving Eq. (7) with $\:\varDelta\:\:=0$, we obtain Eq. (6). The algorithm of I-ELM is presented in Algorithm 2.

#### Algorithm 2.2

**I-ELM**.

The training set and activation function will remain same as in Algorithm 2.1, and the tolerance term will be set to $\:ϵ.$ The I-ELM Algorithm comprises of 2 steps as under:

#### Step (1)

Set and, where .

#### Step (2)

while or, do.

$$\:L=L+1$$

Generate a random number and set $\:{w}_{L}$ and $\:{b}_{L}$.

Calculate the hidden node output matrix $\:{H}_{L}$.

$${\beta\:}_{L}={\left({H}_{L}\cdot{H}_{L}^{\top\:}\right)}^{-1}\left(E\cdot{H}_{L}^{\top\:}\right)$$

$$\:\varvec{E}=\varvec{E}-{\beta\:}_{L}\cdot{H}_{L}$$

end while.

The relevance between hidden nodes and residual determines how many hidden nodes are required to satisfy $\:\parallel E\parallel<\epsilon$. Fewer hidden nodes required if the additional hidden nodes significantly lower the residual; more hidden nodes are required if they very slightly lower the residual. A neural network with an excessively high number of hidden nodes is produced as a result of the existence of hidden nodes that are not very relevant to the residual. A neural network poor learning is caused by having more hidden nodes as system complexity rises, and the ELM system’s performance deteriorates as hidden node count rises. Thus, it is crucial to choose the ideal number of hidden nodes.

Former research on enhancing I-ELM included a technique that creates several hidden nodes in Step 2, assesses their significance to the result, and either adds only high relevant hidden nodes or eliminates irrelevant nodes. By adding a technique to recalculate the output weight and orthogonally modifying the hidden layer output to lower the number of hidden nodes that display a variety of patterns, other studies have also enhanced this system^8– 26^.

## Proposed compact I-ELM approach

I-ELM begins learning by replacing the target value with residuals without initially adding hidden nodes. It then adds the first randomized hidden node and continues to add hidden nodes while updating the residuals. The size of the initial residual is determined by the relevance of the target considering the output of the first random hidden node that is added, and the residual is reduced as more hidden nodes are added. If the output of the first hidden node, which is a random feature map, has a high relevance to the target, then the system begins with a small residual and requires fewer hidden nodes. If the relevance is low, then the system begins with a large residual and requires more hidden nodes. The first hidden node that is added is important for achieving a compact structure, which is a key technique to improve I-ELM.

The Compact I-ELM system proposed in this study does not add the first hidden node randomly. Instead, it adds a hidden node that outputs the pattern that best describes the target in the given data. By first adding the hidden node that is most relevant to the target, the size of the residuals can be greatly reduced, thereby decreasing the number of hidden nodes in the neural network. The pattern that best describes the target in the given data can be found in classical statistics and is a linear regression model. A linear regression model can obtain the regression line that minimizes the residuals from the data.

The proposed Compact I-ELM system uses a linear function as the activation function of the first hidden node, and the input weight and bias are coefficients calculated using the least squares method. This technique also orthogonally transforms the input weights such that each hidden node has a different pattern. If the parameters of the hidden node are set to coefficients calculated by the least square method, then the input of the hidden node becomes an estimate of the linear regression model. The initial residual is the remaining residual after excluding the relationship between the linear feature map and target. Therefore, only a small residual that excludes the part that can be linearly obtained remains. To calculate the linear regression coefficients using the least squares method, the regression equation including a constant term can be expressed in vector form, which is shown as:

8 $$\:\varvec{t}={b}_{1}+{\varvec{w}}_{1}^{T}\varvec{x}+\epsilon$$

Given the data $\:\left\{\right({\varvec{x}}_{\varvec{j}},\:{t}_{j}),\:\:j=1,\cdots\:,\:N\:\}$, if $\:{w}^{*}={[{b}_{1},\:{w}_{1}]}^{\top\:}$and $\:{x}^{*}={[1,\:x]}^{\top\:}$, then $\:{w}^{*}$ can be calculated as:

9 $$\:{\varvec{w}}^{*}={{\left({\varvec{x}}^{*}{\varvec{x}}^{*}\right)}^{-1}\varvec{x}}^{*\top\:}\:\varvec{t}={\varvec{x}}^{+}\varvec{t}$$

Here, $\:{x}^{+}$ is the Moore-Penrose inverse. The constant term of the linear regression coefficient $\:{w}^{*}$ corresponds to $\:{b}_{1}$, and the linear regression coefficient corresponds to $\:{w}_{1}$.

The inputs of the hidden node are $\widehat{t}=b_{1}+{w}_{1}^{\top}\cdot\varvec{x}$ and an estimate of the linear regression model, and the output of the first hidden layer is $\:{H}_{1}=g\left(\widehat{t}\right).$ The first hidden node that is added is a linear hidden node, and subsequent hidden nodes are transformed to have patterns differing from those of the previous hidden nodes before being added. During the process of generating random numbers, it is difficult to generate random numbers that differ from previously generated random numbers. Therefore, a method to transform previously generated random numbers such that they differ from one another is necessary, and Gram-Schmidt orthogonalization is used for this purpose.

The Gram-Schmidt process is a method that converts a finite set of linearly independent vectors in an inner product space into an orthogonal basis. Using a linearly independent set $\:S=\{{v}_{1},\cdots,{v}_{k}\}$ for $\:k\le\:n$, the Gram-Schmidt process generates an orthogonal set $\:S{\prime\:}=\{{u}_{1},\cdots,{u}_{k}\}$ that spans the same $\:k$-dimensional subspace of $\:{R}^{n}$as $\:S$. It finds the orthogonal component by subtracting the projection of $\:{proj}_{u}\left(\varvec{v}\right)$ onto $\:u$ from vector set $\:v$. The Gram-Schmidt process is shown in Eq. (10).

10 $$\begin{aligned}&{\mathbf{u}}_{1}={\mathbf{v}}_{1}\\ &{\mathbf{u}}_{2}={\mathbf{v}}_{2}-{\text{p}\text{r}\text{o}\text{j}}_{{u}_{1}}\left({v}_{2}\right)\\ &{\mathbf{u}}_{3}={\mathbf{v}}_{3}-{proj}_{{u}_{1}}\left({v}_{3}\right)-{proj}_{{u}_{2}}\left({v}_{3}\right)\\&{\mathbf{u}}_{4}={\mathbf{v}}_{4}-{proj}_{{u}_{1}}\left({v}_{4}\right)-{proj}_{{u}_{2}}\left({v}_{4}\right)-{proj}_{{u}_{3}}\left({v}_{4}\right)\\& {\mathbf{u}}_{k}={\mathbf{v}}_{k}-{\sum\:}_{j=1}^{k-1}{proj}_{{u}_{j}}\left({v}_{k}\right) \end{aligned}$$

The orthogonal basis is calculated as $\:{e}_{k}\:=\frac{{u}_{k}}{\parallel{u}_{k}\parallel}$ where $\:{proj}_{u}\left(v\right)=\frac{\langle u,\:u \rangle}{ \langle u,\:u \rangle}u$, and $\: \langle u,\:v \rangle$ is the inner product of vectors $\:u$ and $\:v$. This projection operator projects vector $\:v$ perpendicularly onto the line that vector $\:u$ spans.

As the Gram-Schmidt process changes the inner product of the orthogonalized random numbers, it is necessary to preserve their inner product. Therefore, $\:\parallel {v}_{k}\parallel \ne\:\parallel {u}_{k}\parallel$, which is not appropriate for use as a connection strength. Accordingly, Eq. (11) is used to restore the inner product of the random numbers generated before orthogonalization.

11 $$\mathbf{u}-\frac{\parallel\mathbf{v}\parallel}{\parallel{\mathbf{u}}^{{\prime\:}}\parallel}\times\:{u}^{{\prime\:}}$$

Here,$\:\parallel v \parallel$is$\:\sqrt{{\sum\:}_{i=1}^{n}{v}_{i}^{2}}$and$\:{u}^{{\prime\:}}$is the vector after orthogonalization. By using Eq. (11), $\:\parallel v \parallel= \parallel u \parallel$ can be obtained. The proposed minimized-residual I-ELM algorithm is shown in Algorithm 3.1.

### Algorithm 3.1

**Compact I-ELM**.

The algorithm initiate with setting $\:\aleph\:=\left\{\right({x}_{j},\:{t}_{j}\left)\:\right|\:{x}_{j}\in\:{R}^{n},\:{t}_{j}\in\:R,\:j=1,\cdots\:,\:N\:\},$ which is the training set; $\:g($·) is the activation function; $\:{L}_{max}\:$is the maximum number of nodes; $\:k$ is the number of orthogonalizations; $\epsilon$ is the tolerance term.

### Step (1)

Set L = 1.

Calculate $\:{b}_{1}$ and $\:{\varvec{w}}_{1}$ using Eq. (9) and set them as parameters.

$$\:{\varvec{H}}_{1}={b}_{1}+\sum\:\left({\varvec{w}}_{1}^{T}\varvec{x}\right)\:$$

$$\:{\beta\:}_{1}=\frac{\varvec{T}\cdot\:{\varvec{H}}_{1}^{T}}{{\varvec{H}}_{1}\cdot\:{\varvec{H}}_{1}^{T}}$$

$$\:\varvec{E}=\varvec{T}-{\beta\:}_{1}\cdot\:{H}_{1}$$

**Step (2)**:

while $$L<{L}_{max}\:or\:\parallel E \parallel<\epsilon,\:do$$

$$\:L=L+1$$

Generate a random number and set $\:{w}_{L}$ and $\:{b}_{L}$.

if $$\:1\:<\:k\:\le\:\:n$$ then

$$\:{\varvec{w}}_{L}^{{\prime\:}}={\varvec{w}}_{L}-{\sum\:}_{q=1}^{k-1}{proj}_{{\varvec{u}}_{q}}\left({\varvec{w}}_{L}\right)$$

Where $$\:u=[{w}_{L-1},\dots\:,{w}_{L-k}]$$

$$\:{w}_{L}=|{w}_{L|}/|{w}_{L}^{{\prime\:}}|\times\:{w}_{L}^{{\prime\:}}$$

$$\:k=\:k+\:1$$

else, 

$$\:k\:=\:1$$

end if

$$\:{\beta\:}_{L}=\frac{E\cdot{H}_{L}^{T}}{{H}_{L}\cdot{H}_{L}^{T}}$$

$$\:E=E-{\beta\:}_{L}\cdot{H}_{L}$$

end while.

## Simulation study

In this section, to verify whether the proposed algorithm constructs a neural network with a compact structure, we generate nonlinear functions and perform simulations. The first simulation is used to predict a piecewise linear function (PLF), which is shown in Eq. (12).

12 $$f\left(x\right)=\left\{\begin{array}{cc}-3x,&\:\text{i}\text{f}\:x<-10\\\:x+40,&\:\text{i}\text{f}-10\le\:x<10\\\:-4x+90,&\:\text{i}\text{f}\:x\ge\:10\:.\end{array}\:\:\:\:\right.$$

In total, 3000 values were uniformly generated in the interval [–20, 20] for x. For the training data, noise was added by generating random numbers from a uniform distribution in the interval [–0.2, 0.2], and normalization was performed to obtain values in the range [0, 1].

The second simulation involves the prediction of the function $\:{sin}C$, which is shown in Eq. (13).

13 $$y=\left\{\begin{array}{cc}1,&\:\text{i}\text{f}\:x=0\\\:\:{sin}\left(x\right)/x,&\:\text{i}\text{f}\:x\ne\:0.\end{array}\right.$$

In total, 5000 values were generated from a uniform distribution in the interval [–10, 10], and for the training data, noise was added by generating random numbers from a uniform distribution in the interval [–0.01, 0.01]. The third simulation involves the prediction of a polynomial regression, which is shown in Eq. (14).

14 $$y={x}_{1}+3\times\:{x}_{2}^{2}$$.

Here, 10,000 values were generated from a uniform distribution in the interval [0, 1] for $\:{x}_{1}$ and $\:{x}_{2}$, and noise was added in the training data by generating random numbers from a uniform distribution in the interval [–0.01, 0.01].

For PLF and SinC, the sine function was used as the activation function, and for polynomial regression, the Rectified Linear Unit (ReLu) was used as the activation function. The stopping criterion was set to $\:{L}_{max}=3000$ or $\:e<0.01$, and for polynomial regression, it was set to $\:{L}_{max}=5000$ or $\:e<0.02$. The process was repeated 100 times, and the performance of the neural network was evaluated using the Root Mean Square Error (RMSE) calculated using the number of hidden nodes and noise-free evaluation data. In the training process of the two algorithms, to eliminate the influence of the randomly generated numbers on the structure and performance of the neural network, the parameter values of the random hidden nodes were set to share the random numbers generated at the time at which each hidden node was added.

Table 1 shows the results of the number of hidden nodes obtained for comparing the neural network structures, and Table 2 shows the performances of the neural networks obtained using the evaluation data. The results indicate that for PLF, the I-ELM method required 689.5 hidden nodes, whereas Compact I-ELM required 152 hidden nodes. For the SinC function, the I-ELM method required 543.5 hidden nodes, while Compact I-ELM required 333 hidden nodes. For polynomial regression, the I-ELM method required 1,264 hidden nodes, while Compact I-ELM required 174.5 hidden nodes. In this study, Compact I-ELM was able to achieve the same performance using a neural network with fewer hidden nodes than the existing method. Therefore, a neural network with a compact structure was constructed.

**Table 1:** Complexity comparison results of the different algorithms.

Algorithms	PLF		$\:SinC$		Polynomial	
	Median	Q1–Q3	Median	Q1–Q3	Median	Q1–Q3
I-ELM	689.5	(310.5–1228.8)	543.5	(156.0–958.5)	1264.0	(472.8–3813.5)
Compact I-ELM	152.0	(92.8–322.3)	333.0	(124.0–783.8)	174.5	(133.8–211.0)

**Table 2:** Performance comparison of the different algorithms: RMSE in noise-free data.

Algorithms	PLF		$\:SinC$		Polynomial	
	Mean	Std.	Mean	Std.	Mean	Std.
I-ELM	0.010	0.001	0.008	0.001	0.021	0.003
Compact I-ELM	0.010	0.001	0.008	0.001	0.020	0.003

## Performance evaluation

In this section, to verify whether the proposed algorithm is able to provide a compact neural network structure, we evaluate and compare this system with the existing I-ELM, orthogonal node (which orthogonalizes and adds hidden nodes), first linear node (which adds linear regression nodes), and Compact I-ELM (which combines the two preceding methods) algorithms. A total of 13 benchmark datasets were used to solve the regression problem, and the characteristics of each dataset are presented in Table 3. As the purpose of this study is to examine how the neural network structure is determined, cross-validation was not performed and the data was only divided into training and testing datasets at a ratio of 70 to 30%, respectively. Random numbers were generated from a uniform distribution in the interval [0, 1] for the input weights and bias values of all hidden nodes in the neural network, and the sigmoid function was used as the activation function of the hidden nodes. In the training process of each algorithm, random numbers were generated differently to configure the random hidden nodes. Because this process can impact the performance of the neural network, we set each algorithm to share the random numbers generated during the process of adding each hidden node. The maximum number of hidden nodes ($\:{L}_{max}$) was set to 5000, the performance was measured by the RMSE, and $\:\epsilon\:$ was set to be 0.0005-smaller than the RMSE of the linear regression analysis. For each dataset, the process was repeated 100 times and the average number of hidden nodes and average RMSE of the test data were presented. All simulations were implemented using Python 3.7.

Table 4 shows the average number of hidden nodes required to satisfy the stopping criterion for the neural network. The first linear node algorithm and the Compact I-ELM algorithm, which combines the first linear node and orthogonal node, constructed neural networks with fewer hidden nodes compared to the existing I-ELM system. The proposed algorithm constructed a more compact neural network with the same performance as the existing I-ELM, and because the structure of the proposed system is more compact, the learning time was reduced. However, for some benchmark data, it did not reach the required RMSE and obtained the maximum number of hidden nodes. This indicates that it is not possible to construct a neural network with a better performance than that of a linear regression model.

Table 5 shows the performance of the proposed model on the test dataset. The proposed model exhibited similar or even better performance compared to the existing model. This indicates that a compact neural network with fewer nodes can effectively solve the problem with the same level of performance as the traditional I-ELM. It also implies that the compact neural network allows for faster learning and computations, making it efficient.

**Table 3:** Characteristics of the benchmark datasets.

Datasets	No. of features	Sample	Training	Testing
Abalone	9	4,177	2,924	1,253
Auto MPG	7	392	274	118
Auto-Price	16	159	111	48
Bank	33	4,500	3,150	1,350
Boston housing	14	394	276	118
California housing	10	20,433	14,303	6,130
Computer activity	22	8,192	5,734	2,458
Delta ailerons	6	7,129	4,990	2,139
Delta elevators	7	9,517	6,662	2,855
NASA airfoil	6	1,503	1,052	451
Machine-CPU	7	209	146	63
Servo	5	167	117	50
Wisconsin breast cancer	33	194	136	58

**Table 4:** Complexity comparison of the different algorithms: mean number of hidden nodes.

Datasets	I-ELM	Orthogonal nodes	First Linear node	Compact I-ELM
Abalone	4926.1	4869.8	77.5	78.8
Auto MPG	482.7	478.7	8.6	8.8
Auto-Price	874.1	860.7	14.4	14.7
Bank datasets (bank32NH)	2218.3	2114.5	21.4	25.9
Boston Housing	929.1	915.6	16.2	20.4
California Housing	5000.0	5000.0	5.7	5.5
Computer Activity	1577.5	1292.6	15.2	16.9
Delta ailerons	4009.9	4039.9	2916.3	3084.2
Delta Elevators	5000.0	5000.0	5000.0	5000.0
Machine-CPU	111.3	99.5	13.0	16.9
NASA airfoil	858.5	929.6	58.4	60.4
Servo	355.2	340.2	3.2	3.1
Wisconsin Breast Cancer	1904.6	1862.0	4.9	4.7

Considering the Delta Elevators dataset, the proposed algorithm reached the maximum number of hidden nodes as a result of regression analysis.

**Table 5:** Performance comparison of the different algorithms: test scores.

Datasets	I-ELM	Orthogonal nodes	First Linear node	Compact I-ELM
Abalone	0.0831	0.0830	0.0849	0.0849
Auto MPG	0.0985	0.0983	0.0968	0.0968
Auto-Price	0.1087	0.1084	0.1062	0.1062
Bank datasets (bank32NH)	0.1251	0.1251	0.1209	0.1209
Boston Housing	0.1041	0.1036	0.1028	0.1028
California Housing	0.1397	0.1396	0.1401	0.1401
Computer Activity	0.0949	0.0945	0.0920	0.0920
Delta ailerons	0.0398	0.0398	0.0396	0.0396
Delta Elevators	0.0566	0.0566	0.0562	0.0562
Machine-CPU	0.0420	0.0415	0.0441	0.0440
NASA airfoil	0.1315	0.1316	0.1331	0.1331
Servo	0.1473	0.1468	0.1462	0.1462
Wisconsin Breast Cancer	0.2574	0.2579	0.2893	0.2893

## Conclusion and recommendation

This study proposed a compact algorithm as a method for constructing compact neural networks using I-ELM. The compact algorithm adds a linear node as the first hidden node and then orthogonalizes subsequent hidden nodes to exhibit different patterns from the existing hidden nodes. The first hidden node handles the most important patterns in the data, and subsequent hidden nodes are created to explain patterns that were not explained by previous hidden nodes. The proposed method can not only construct a compact neural network, but it was also demonstrated that problems that can be solved with a linear regression model do not require the use of a complex neural network. The proposed method was shown to be able to construct a compact neural network using benchmark data. The simplicity of I-ELM can be utilized to reduce the trial-and-error process associated with determining the structure of neural networks and reduce research time.

The study results are limited by the problem of determining the optimal stopping criterion and is only considered from the perspective of hidden nodes. While I-ELM has the advantage of online learning and the ability to adapt to changing data, it has many problems, such as overfitting and generalization problems, limited support for different types of data, and sensitivity to parameter setting. Future research can be done by considering the above-mentioned problems to increase the scope of the Compact I-ELM Approach. Further, we create a compact neural network structure rather than applying it, we proposed a method to solve the problem of determining the existing model structure. When determining the model structure, if the weights are determined by the random numbers and the method proposed in this study is applied, the performance will be the same, but the model structure will be smaller. Thus, in the future, it will be able to reduce resources and obtain the same performance with a compact structure if applied to current deep learning models.

Moreover, of all types of methods, Compact I-ELM has phenomenal limitations particularly when constructing large and diverse neural networks. For instance, it can suffer from memory and computational complexity troubles when big data structures are being searched, used or analyzed, which may hamper real-time efficiency. The specifics of how to train deep networks in regards to their parts such as activation functions and layers also serve as a significant limitation to Compact I-ELM to various forms of neural networks. This measure show that Compact I-ELM cannot sustain most of applying practice, and it is needed critical development in this direction to make it various. Thus, to make it more robust and adaptable to a wider range of applications, further experimentation and research is required.

## Data Availability

The data used in this study were public data provided by the LIACC - Artificial Intelligence and Computer Science Laboratory. The data sets were downloaded from the following website: https://www.dcc.fc.up.pt/~ltorgo/Regression/DataSets.html. The data are provided in a format ready for use with the RT tree induction system and can also be utilized with Cubist, Mars, and CART through available scripts. Detailed information on the format of the files included for each problem can be found on the repository’s webpage.
