# Author Correction: Improving I-ELM structure through optimal addition of hidden nodes: Compact I-ELM

**DOI:** 10.1038/s41598-025-88175-1

**Published:** 2025-02-12

**Authors:** Sunghyo Seo, Jongkwon Jo, Muhammad Hamza, Youngsoon Kim

**Affiliations:** 1 Department of Information and Statistics, Gyeongsang National University, 501, Jinju-Daero, Jinju-Si, Gyeongsangnam-Do Republic of Korea; 2 Department of Bio & Medical Big Data (BK21 Four Program), Gyeongsang National University, 501, Jinju-Daero, Jinju-Si, Gyeongsangnam-Do Republic of Korea

## Introduction

The original version of this Article contained an error in the acknowledgement and funding section. The Grant Number of the Institute of Information and Communication Technology Planning and Evaluation (IITP) was incorrectly written as IITP -2024-RS-2022-00156360.

This now reads as IITP-2024-RS-2022-00156409.

The original Article has been corrected.

